# Genome-Wide Identification and Expression Analysis of the Xyloglucan Endotransglucosylase/Hydrolase Gene Family in *Manihot esculenta*

**DOI:** 10.3390/genes17060613

**Published:** 2026-05-29

**Authors:** Hao Ju, Jing Chu, Qing Xie, Abduaziz Abduvasikov, Yu Wang, Xingyu Jiang

**Affiliations:** 1 National Center of Technology Innovation for Saline-Alkali Tolerant Rice, College of Coastal Agricultural Sciences, Guangdong Ocean University, Zhanjiang 524088, China; 2 Hainan Key Laboratory for Biotechnology of Salt Tolerant Crops, Institute of Tropical Crops, Hainan University, Haikou 570228, China; 3 Department of International Cooperation, Transformation and Strategic Development, Tashkent State Agrarian University, Tashkent 100140, Uzbekistan

**Keywords:** cassava, *MeXTH* gene family, gene expression analysis, subcellular localization

## Abstract

**Background**: Xyloglucan endotransglucosylase/hydrolase (XTH) acts as a key cell wall-modifying enzyme and contributes to plant stress resilience. This study aimed to identify the *MeXTH* gene family in cassava and characterize its potential functions in abiotic stress adaptation. **Method**: A full set of bioinformatic analyses was performed, including phylogeny, gene structure, conserved motifs, chromosomal localization, synteny, promoter cis-elements and subcellular localization. Expression patterns were examined by quantitative real-time PCR (qRT-PCR). **Results**: Forty-two *MeXTH* genes were identified and distributed on 14 chromosomes, encoding proteins with conserved Glyco_hydro_16 (Glycoside hydrolase family 16) and XET_C (Xyloglucan endotransglycosylase C-terminal domain) domains. Genes were clustered into four subfamilies with similar structures. Synteny was closer between cassava and dicots than monocots. Twenty-four stress-, hormone- and light-related cis-elements were detected. Ten *MeXTH* genes showed obvious differential expression under stress, and most proteins were located in the cell wall. **Conclusions**: The *MeXTH* gene family is structurally conserved and can serve as a readout of abiotic stress in cassava. These results provide a theoretical basis for molecular breeding aimed at enhancing stress resistance in cassava.

## 1. Introduction

Cassava (*Manihot esculenta*) belongs to the Euphorbiaceae family and is a dicotyledonous short-day tropical crop [1]. It is the sixth major food crop in the world after corn, rice, wheat, potatoes and soybeans [2]. Cassava serves not only as a strategic crop critical to global food security and industrial advancement but also as a pivotal model system for genetic research and the application of modern breeding technologies [3]. It serves multiple socioeconomic functions: as a primary food source, animal feed ingredient, and industrial raw material. The starch content in cassava root is relatively high, and it is called “the king of starch”, and sustains the dietary needs of approximately 800 million people in developing regions, particularly sub-Saharan Africa and South Asia. Moreover, cassava starch is increasingly utilized as a feedstock for bioethanol production, contributing to renewable energy initiatives [2,4,5]. As a representative tuberous root crop, cassava also serves as a crucial system for testing and applying modern breeding techniques, including marker-assisted selection, genome editing, transgenic engineering, and genomic selection. Progress in cassava functional genomics and molecular breeding not only accelerates the genetic improvement of yield, quality, and stress resistance, but also provides valuable references for the genetic enhancement of other tuber and root crops [6]. Abiotic stress severely limits the yield and quality of cassava, causing huge economic losses to agricultural production. High-yield and high-efficiency cultivation of cassava has become the main research direction at present. Consequently, enhancing yield stability and resource-use efficiency through genetic improvement has emerged as a central objective in contemporary cassava research [7].

The cell wall structure of plants is an indispensable component of plants, mainly composed of substances such as cellulose, hemicellulose, pectin and lignin [8], and it is also the primary cellular structure for sensing external stress signals. In addition to maintaining the connections between cells, controlling cell elongation, maintaining cell morphology and determining the strength of cell walls, it also plays a crucial role in cell differentiation, recognition and cell signal transduction, etc. The enzymes encoded by the Xyloglucan endotransglucosylase/hydrolase (XTH) gene family play a crucial role in plant cell wall remodeling [9]. XTH has both XET (Xyloglucan Endotransglycosylase) and XEH (Xyloglucan Endohydrolase) activities, but the activities of different members vary. XET plays a role in modifying the cellulose–xyloglucan complex structure in the cell wall by cutting off the xyloglucan chain and reconnecting it to other chains, while XEH directly hydrolyzes the xyloglucan chain to promote its degradation and realize the dynamic reconstruction of the cell wall network [10]. The synergistic action of the XET and XEH domains determines the catalytic activity and hydrolytic properties of the XTH protein. As part of the *XTH OsXTH11* combines two kinds of activity, and *OsXTH19* and *OsXTH20* only show XEH activity [11], the tomato *SlXTH5* is given priority to XET activity [12], and the XTH protein function differentiation makes plants to meet the needs of different development or the environment.

In recent years, studies have shown that the XTH protein is involved in multiple growth and development processes of plants, such as root elongation, leaf vein differentiation, flower growth and development, fruit ripening, petal senescence, etc. Overexpression of *Arabidopsis thaliana AtXTH18/19/20* can promote hypocotyl elongation and enhance cell wall plasticity, while inhibition of expression delays the growth of *A. thaliana* [13]. In sweet cherry, *PavXTH14* and *PavXTH15* directly promote fruit softening by degrading cell wall components such as hemicellulose and pectin [14]. In the early stage of tomato fruit development, the highly expressed *SlXTH12* may maintain the integrity of the cell wall structure, while the highly expressed *SlXTH5* and *SlXTH8* in the maturation stage and related to fruit softening are positively regulated by ethylene [15]. Apple *MdXTH10* also depends on the ethylene signal, and its overexpression accelerates tomato fruit softening and activates the key genes *ACS2* and *ACO1* for ethylene synthesis [16]. These findings suggest that the XTH protein plays a key role in plant growth and development, and its possible role in cassava’s abiotic stress response has not been fully investigated. Therefore, 42 *MeXTH* genes have been identified throughout the cassava genome. Their phylogeny, gene structure, conserved motifs, chromosome distribution, intraspecies and interspecific collinearity and cis-acting elements were comprehensively analyzed, and their tissue-specific expression was analyzed by quantitative real-time PCR (qRT-PCR) to explore their expression patterns under abiotic stress. Subcellular localization of MeXTH protein was also investigated, which laid the foundation for elucidation of the *MeXTH*-mediated stress regulation mechanism in cassava.

## 2. Materials and Methods

### 2.1. Plant Materials and Processing

The cassava used in the experiment (Cassava No. 9 from South China, grown at the National Center of Technology Innovation for Saline-Alkali Tolerant Rice) was provided by the Institute of Tropical Crops. Cassava seedlings were grown in MS Modified Medium (with vitamins, Sucrose, Agar) (PM10121-307, Coolaber, Beijing, China). Acquisition of cassava tissue culture plantlets: The stems of the Cassava No. 9 seedlings grown in the greenhouse, which were evenly growing towards the middle, were selected. The leaves and petioles on the nodes were removed, and then they were rinsed with running water for about 1 h for preliminary disinfection. The seedlings were then disinfected again using sterile tools, sterile distilled water, and 75% alcohol in a clean bench, and the stem segments about 1 cm long were taken and inserted into the MS medium. Finally, they were placed in a constant temperature and light incubator (26 °C, 15,000 lx) for about 20 days. The cassava tissue culture seedlings were acclimated to the environment for about 20 days and then transplanted into pot culture boxes for another 10 days of growth. The levels of each abiotic stress were set according to preliminary pilot experiments as well as literature reports from related studies on cassava or other model plants, in order to ensure appropriate and effective stress treatments [17,18,19,20]. In the quantitative real-time PCR (qRT-PCR) analysis, the following treatments were selected: drought stress with 20% PEG (polyethylene glycol), salt stress with 200 mmol/L NaCl, high-temperature stress at 42 °C, and low-temperature stress at 4 °C. Sampling was conducted at different time points (drought stress was sampled at 1 h, 3 h, 6 h, and 12 h after 20% PEG treatment, salt stress was sampled at 6 h, 12 h, 24 h, and 36 h after 200 mmol/L NaCl treatment, high-temperature stress was sampled at 3 h, 6 h, 12 h, and 36 h after 42 °C treatment, low-temperature stress was sampled at 3 h, 6 h, 12 h, and 24 h after 4 °C treatment). Root, stem, and leaf samples were first washed with tap water, then rinsed with distilled water, thoroughly dried, and immediately frozen with liquid nitrogen before being stored in a −80 °C ultra-low-temperature refrigerator for RNA extraction.

### 2.2. Identification of MeXTH Gene Family and Physicochemical Property Analysis

Download the Hidden Markov Models (HMMs) of the XTH conserved domains (PF00722 and PF06955) from the Pfam database (http://pfam.xfam.org/ (accessed on 15 September 2024)). Subsequently, perform BLAST (v2.16.0) and HMMER (v3.4) searches on the cassava genome. Use the BLASTP program (with an E-value threshold set to <e^−10^ and sequence similarity >30%) to identify homologous *XTH* genes in cassava and further verify them using the SMART database(http://smart.embl.de/ (accessed on 15 September 2024)). Information including amino acid length, chromosomal location and gene ID of MeXTH gene family members were extracted from GFF3 annotation files using TBtools (v2.322) [21]. The physicochemical properties, molecular weight, and isoelectric point of MeXTH family proteins were predicted using ExPasy (v3.0) [22] (http://web.expasy.org/compute_pi/ (accessed on 20 October 2024)). Subcellular localization of MeXTH proteins was predicted using Cell-PLoc 2.0. [23] (http://www.csbio.sjtu.edu.cn/bioinf/Cell-PLoc-2/ (accessed on 20 October 2024)).

### 2.3. Conserved Motifs, Protein Domains and Gene Structure of MeXTH

The conserved motifs of the MeXTH gene were predicted using the online software MEME suite (v5.5) [24] (https://meme-suite.org/meme/tools/meme (accessed on 15 September 2024)). The MEME results were visualized using the MEME/MAST Motif Pattern Redrawer module in TBtools (v2.322). Protein domain structures were visualized using the Visualize Domain Pattern module in TBtools (v2.322). We visualized the gene structures of MeXTH family members using the Gene Structure Visualization module in TBtools (v2.322).

### 2.4. Chromosomal Localization and Phylogenetic Tree Analysis of the MeXTH Proteins

Based on the cassava genome information provided by the Tropical Crop Omics Database (https://ngdc.cncb.ac.cn/tcod/genes/ (accessed on 20 October 2024)), combined with the chromosomal location information of MeXTH proteins, through TBtools (v2.322) software, fine mapping of the members of this gene family was performed. Using the Clustal W module of MEGA11.0 software [25], under the default settings (Gap open −2.90, Gap Extend 0), multiple sequence alignments of the amino acid sequences of *XTH* gene family members in *A. thaliana* and cassava were conducted. Based on the alignment results, a phylogenetic tree was constructed using the neighbor-joining method [26] (neighbor-joining, NJ) in MEGA11.0 software, with the bootstrap value set at 1000 [27]. Phylogenetic trees were visualized using the Gene Structure View (Advanced) module in TBtools (v2.322).

### 2.5. Collinearity Analysis of the MeXTH Gene Family

*A. thaliana* (TAIR10), rice (v7.0), and tomato (SL3.0) genome sequences and annotation files were downloaded from the Phytozome v13 website (https://phytozome-next.jgi.doe.gov/ (accessed on 20 October 2024)). Barley genome and annotation files (GCF_904849725) were retrieved from the NCBI database (https://www.ncbi.nlm.nih.gov/ (accessed on 20 October 2024)). Collinearity relationships among *XTH* genes in cassava, as well as between cassava and *A. thaliana*, rice, tomato, and barley, were analyzed using MCScanX (v1.0) software [28] with default parameters. The identified collinear *XTH* gene pairs were visualized using TBtools (v2.322).

### 2.6. Analysis of Cis-Elements in MeXTH Promoters

A 2000 bp sequence upstream of the coding region of each *MeXTH* gene was retrieved. Cis-elements in the MeXTH gene promoters were predicted using the PlantCARE online database [29] (https://bioinformatiCs.psb.ugent.be/ (accessed on 20 October 2024)). Visualization of cis-elements related to stress responses, plant growth and development, plant hormone responsiveness, and light responses was performed using the Basic Biosequence View and HeatMap modules in TBtools (v2.322), and the distribution patterns were summarized.

### 2.7. Expression Profiling Based on RNA-Seq Data

In order to study the spatial expression map of the *MeXTH* gene, the RNA-seq data (GEO dataset: GSE82279 and GSE156638) from the NCBI website (https://www.ncbi.nlm.nih.gov/bioproject/ (accessed on 20 October 2024)) was utilized to analyze the expression patterns of the *MeXTH* gene in different tissues as well as after 7% (w/v) PEG-induced osmotic stress and 50 uM ABA (abscisic acid) treatments, including petioles, stems, roots, storage roots, fibrous roots, leaves, midveins, lateral buds, shoot apical meristems, apical meristems, root apical meristems, somatic embryos and friable embryogenic calli. The reference genome index of cassava was established by the kallisto (v0.51.1) tool [30], and then the expression data was quantified. The heatmap of the spatial expression map of the *MeXTH* gene was constructed using TBtools (v2.322).

### 2.8. Quantitative Real-Time PCR (qRT-PCR) Analysis of the MeXTH Genes

The total RNA of cassava was extracted using the CTAB method [31], and the nucleic acid content was quantified using the Nano-300 micro spectrophotometer(Hangzhou Allsheng Instruments Co., Ltd., Hangzhou, China). It was confirmed that the samples met the experimental standards (A260/A280 = 1.8–2.1). Subsequently, the first-strand cDNA synthesis kit (MightScript Plus First Strand cDNA Synthesis Master Mix, Sangon Biotech (Shanghai) Co., Ltd., Guangzhou, China) was used for reverse transcription to synthesize cDNA. The obtained cDNA products were aliquoted and long-term frozen at −80 °C in an ultra-low-temperature refrigerator. The qRT-PCR primers for the *MeXTH* gene were specifically designed through the NCBI website Primer-Blast (Primer sequence information is shown in Supplementary Materials), and the primer synthesis was completed by Sangon Biotech (Shanghai) Co., Ltd. (Guangzhou, China). Quantitative analysis was performed using the Bio-Rad CFX Real-Time PCR System(Bio-Rad Laboratories, California, USA). The qRT-PCR reaction mixture (20 uL total volume) consisted of the following components: 10 μL of 2 × SYBR Green Pro Taq HS Premix (Accurate Biotechnology Co., Ltd., Beijing, China. final concentration: 1×), 0.4 μL each of forward and reverse primers (final concentration: 0.2 μM), 1 μL of cDNA template (≤100 ng per reaction), and 8.2 μL of nuclease-free water. The reaction program was set as 95 °C for 30 s pre-denaturation, followed by 40 cycles, each cycle including 5 s of denaturation at 95 °C, 20 s of annealing at 60 °C, and 30 s of extension at 72 °C. The expression level was standardized using cassava actin [32] as the internal reference gene. Three independent biological replicates were set for each treatment, and the relative expression level was calculated using the 2^−ΔΔCt^ method [33]. GraphPad Prism (v10.1.2) was used to draw the charts. Differences were considered significant at *p* < 0.05, and the data are expressed as the mean ± standard error of three independent biological replicates.

### 2.9. Subcellular Localization Analysis

Using the homologous recombination technique [34], the full-length coding sequence of the *MeXTH* gene was cloned into the 35S:EGFP plant expression vector pSCZ, forming a 35S:MeXTH-EGFP fusion expression vector. The aforementioned recombinant plasmid was transformed into Agrobacterium GV3101 using the Agrobacterium transformation method [35]. The positive cloning strains were then transiently transformed into tobacco leaves through the tobacco leaf transient transformation system, and fluorescence was detected using a laser confocal microscope under the excitation wavelength of GFP (488 nm/505–550 nm).

## 3. Results

### 3.1. Identification and Physicochemical Property Analysis of the MeXTH Gene Family

Table S1 shows that the amino acid length of 42 MeXTH proteins ranges from 262 to 348 aa, and the number of amino acids encoded by MeXTH2 is the least, which is 262. Meanwhile, MeXTH40 encodes the most amino acids, with 348. The molecular weights of the MeXTH gene family members range from 29.61 to 40.10 kDa, and their isoelectric points range from 4.55 to 9.40. The total average hydrophilicity of the MeXTH proteins is negative, indicating they are hydrophobic proteins. A total of 24 of the proteins have an unstable coefficient of less than or equal to 40, which belongs to stable proteins, and 25 of the proteins have an isoelectric point less than 7, which belongs to acidic proteins. The subcellular localization prediction shows that 20 MeXTH proteins are located in the cell wall, and 22 proteins are present in both the cell wall and the cytoplasm. There are significant differences in relative molecular mass, protein length, etc., among the members of the MeXTH gene family, revealing the diversity of the MeXTH proteins.

### 3.2. Analysis of the Structure, Conserved Motifs, and Domains of the MeXTH Genes

**Figure 1:**
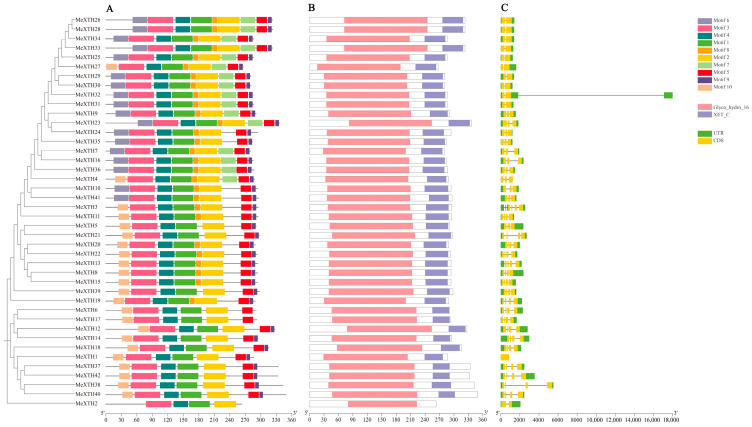
(**A**) The conserved motifs of MeXTH. (**B**) The protein domains of MeXTH. (**C**) The gene structure of MeXTH.

From the perspective of the conserved motifs of the MeXTH gene (Figure 1A), the majority of the MeXTH gene family contains nine conserved motifs. The types and quantities of conserved motifs among the members of these gene families vary. There are 12 members of the MeXTH gene family that contain eight motifs, and the arrangement order of these eight motifs remains consistent, all starting from Motif 10 and ending at Motif 9; there are 16 members of the MeXTH gene family containing nine motifs, all starting from Motif 6 and ending at Motif 9. From the perspective of the MeXTH gene structure (Figure 1B), except for MeXTH1, which has no introns, the other proteins contain at least two or more introns and exons, indicating that they have similar gene structures. From the perspective of the MeXTH protein domain (Figure 1C), except for the MeXTH2 protein, which only contains the typical Glyco_hydro_16 (PF00722) catalytic domain, the rest of the MeXTH proteins all have the typical Glyco_hydro_16 (PF00722) catalytic domain and the conserved XET_C (PF06955) domain. The similar conserved motifs, gene structures, and protein domains of the MeXTH proteins suggest that they may have similar functions.

### 3.3. Phylogenetic Tree Analysis and Chromosomal Localization of the MeXTH Proteins

**Figure 2:**
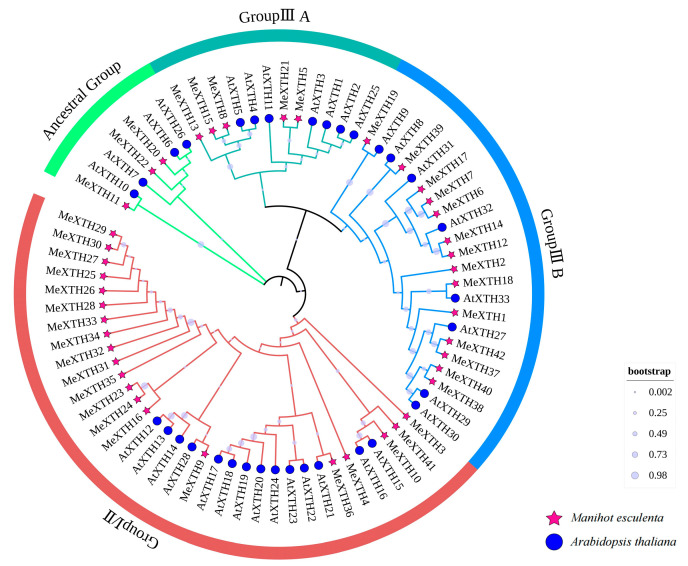
Phylogenetic tree analysis of XTH proteins of *M. esculenta* and *A. thaliana*.

**Figure 3:**
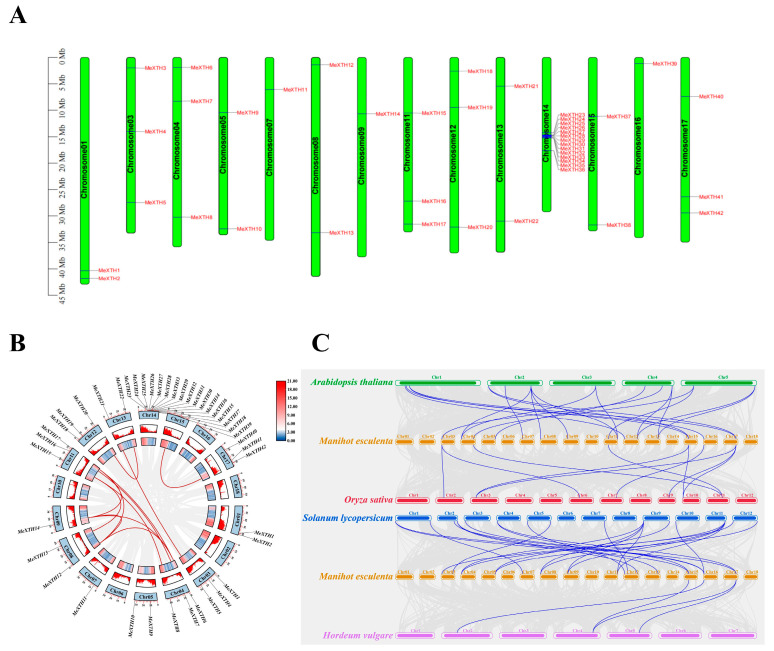
(**A**) Chromosome localization of the MeXTH gene family. (**B**) Co-linearity analysis of *XTH* within cassava species. The gray area represents the co-linearity blocks in the cassava genome, and the red lines between the *MeXTH* genes indicate the segment duplication events occurring within the cassava *MeXTH* gene family. (**C**) Co-linearity analysis of *XTH* genes between cassava and *A. thaliana*, rice, tomato, and barley. The gray lines represent the co-linearity blocks between the *M. esculenta* and the genomes of *A. thaliana*, rice, tomato, and barley. The blue lines indicate the homologous gene pairs in the genomes of *M. esculenta*, *A. thaliana*, rice, tomato, and barley.

Each MeXTH was named and grouped based on the closeness of their kinship with the AtXTH gene family, and a phylogenetic tree was constructed, which was divided into four subfamilies: Ancestral Group, Group I/II, GroupIIIA and GroupIIIB. The phylogenetic tree (Figure 2) reveals that Group I/II contains the largest number of members of the MeXTH gene family, with 20 MeXTH proteins and 14 AtXTH proteins. The Ancestral Group only has MeXTH11, MeXTH21, MeXTH22 and four AtXTH proteins. Each subfamily includes XTH proteins from *A. thaliana* and cassava. The cassava MeXTH protein has a high degree of consistency with the four subfamilies of the AtXTH family in *Arabidopsis*, indicating that the XTH protein of cassava and *A. thaliana* have a similar genetic relationship. According to the distribution of genes on chromosomes (Figure 3A), 42 *MeXTH* genes were unevenly distributed on 14 chromosomes in the cassava genome, among which chromosome 14 showed a high aggregation phenomenon, and 14 gene family members were distributed. On chromosomes 3, 4, 10, 11, 12 and 17, three *MeXTH* genes are located. On chromosomes 7, 9, and 16, there is only one *MeXTH* gene.

### 3.4. Intraspecific and Interspecific Collinearity Analysis of the MeXTH Gene Family

To investigate the repetitive events of the *MeXTH* gene, an intraspecific gene collinearity analysis was conducted (Figure 3B). No tandemly repeated genes of the *MeXTH* gene were found in the cassava chromosomes. Twelve pairs of *MeXTH* genes were generated by fragment duplication and were distributed on nine chromosomes, indicating that some *MeXTH* genes might be produced by gene duplication, and fragment duplication played an important role in the evolution of the *MeXTH* gene family. To further explore the evolutionary relationship of the *MeXTH* gene family, an interspecific gene co-linearity analysis was conducted between cassava and *A. thaliana*, rice, tomato, and barley (Figure 3C). The cross-species gene co-linearity analysis results showed that 38 *MeXTH* genes were identified to have significant co-linearity relationships with other species. Among them, 15 cassava *MeXTH* genes had co-linearity with *A. thaliana AtXTH* genes. These cassava *MeXTH* genes were distributed on chromosomes 3, 4, 5, 7, 8, 9, 11, 12, 13, 15, 16, and 17, while the corresponding *A. thaliana* genes *AtXTH* were distributed on five chromosomes. Additionally, six *MeXTH* genes had co-linearity with seven *OsXTH* genes of rice, 15 *MeXTH* genes had co-linearity with the tomato *SlXTH* gene, and three *MeXTH* genes had co-linearity with three *HvXTH* genes of barley. Based on the number of co-linearity genes of the five species, it can be seen that the co-linearity relationship between cassava and dicotyledonous plant *A. thaliana* is stronger than that between monocotyledonous plant rice, and the co-linearity relationship between cassava and dicotyledonous plant tomato is stronger than that between monocotyledonous plant barley. Therefore, the evolutionary relationship between cassava and *A. thaliana* and tomato is closer than that between rice and barley.

### 3.5. Analysis of Cis-Acting Elements in the Promoter Regions of the MeXTH Gene Family

**Figure 4:**
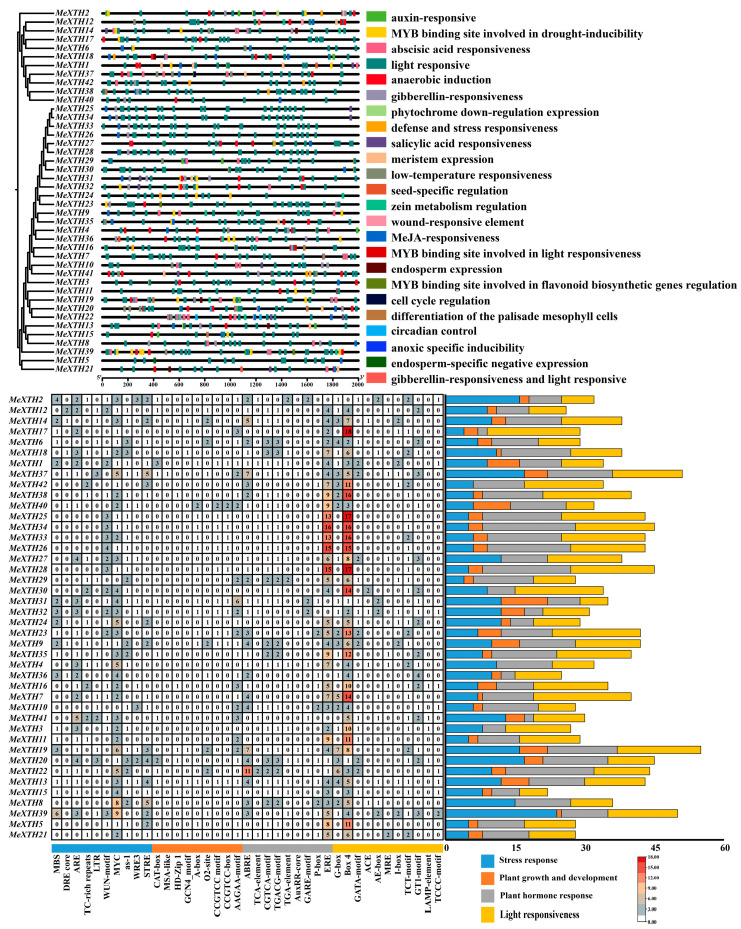
The distribution and quantity of cis-acting elements in the promoter regions of 42 *MeXTH* genes.

The analysis results of cis-acting elements (Figure 4) showed that there were 24 different cis-acting elements in the *MeXTH* gene promoter region, some of which were associated with abiotic stresses (such as drought, low temperature, and defense response), meristem expression, circadian rhythm control, cell cycle regulation, and light response. These cis-acting elements can be divided into four main groups according to their biological functions: light response elements (such as Box 4, G-Box, TCT-motif, etc.), hormone response elements (such as ABRE, ERE, TGACG-motif, etc.), environmental stress response elements (such as MBS, LTR, TC-rich repeats, etc.), and growth and development regulatory elements (such as CAT-box, GCN4_motif, O2-site, etc.). Among them, the Box 4 element is the most numerous, with 331 elements in 41 *MeXTH* genes. The hormone response element ERE has 229 elements, and 19 *MeXTH* genes contain MYB binding sites involved in drought-induced stress (MBS), and 14 genes contain low-temperature response elements (LTR), etc. These results suggest that the *MeXTH* gene may play a significant role in plant growth, development and stress response.

### 3.6. Expression Profiles of MeXTH Genes in Different Cassava Tissues and Different Treatments Based on RNA-Seq Data

**Figure 5:**
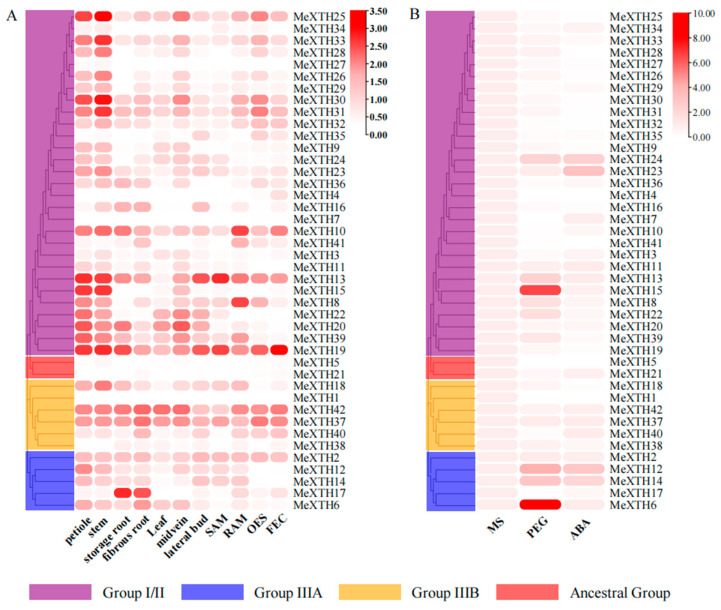
Analysis of tissue-specific expression of the *MeXTH* gene family in cassava. (**A**) Expression profile of *MeXTH* genes in different cassava tissues. (**B**) Expression profile of *MeXTH* genes under different treatments. SAM: shoot apical meristem. RAM: root apical meristem. OES: somatic embryos. FEC: friable embryogenic callus. MS: MS medium. PEG: polyethylene glycol; ABA: abscisic acid.

Transcriptome analysis revealed that 42 *MeXTH* genes displayed detectable transcript abundance across eleven cassava tissues, with pronounced inter-gene and tissue-specific expression divergence. Specifically, *MeXTH13/15/19/20/25/30/39* exhibited significantly elevated expression in petioles (Figure 5A). In storage roots, *MeXTH10/13/17/19/20/42* showed relatively high transcript levels. In stems, *MeXTH13/15/19/25/30/31/33* were highly expressed. *MeXTH20/25/30/33/42* displayed preferential accumulation in midveins. Notably, *MeXTH13* and *MeXTH19* showed dominant expression in shoot apical meristems, whereas *MeXTH8* and *MeXTH10* exhibited pronounced transcript enrichment specifically in root apical meristems. Under 7% (w/v) PEG-induced osmotic stress, *MeXTH6/12/13/14/15/23/24* showed pronounced transcriptional induction (Figure 5B). Similarly, under 50 uM ABA treatment, *MeXTH12/14/23/24* exhibited significantly upregulated expression. Collectively, these spatially resolved expression patterns strongly support functional diversification among *MeXTH* family members during cassava development.

### 3.7. Time-Specific Analysis of MeXTH Gene Expression Under Abiotic Stress

**Figure 6:**
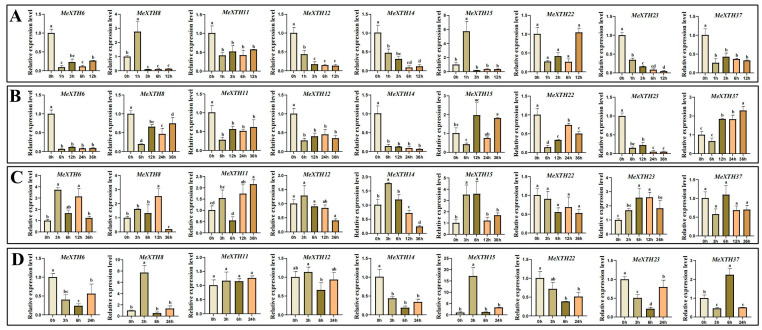
Expression analysis of the *MeXTH* gene in the roots under abiotic stress conditions. (**A**) PEG treatment. (**B**) Salt treatment. (**C**) Heat treatment. (**D**) Cold treatment. Each bar represents the subject mean of three replicates, and error bars represent standard deviation (SD). Lowercase letters indicate differences significant at the 0.05 level.

**Figure 7:**
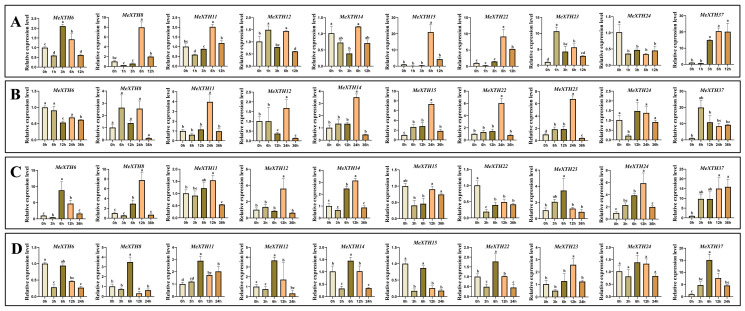
Expression analysis of the *MeXTH* gene in the stem under abiotic stress conditions. (**A**) PEG treatment. (**B**) Salt treatment. (**C**) Heat treatment. (**D**) Cold treatment. Each bar represents the subject mean of three replicates, and error bars represent standard deviation (SD). Lowercase letters indicate differences significant at the 0.05 level.

**Figure 8:**
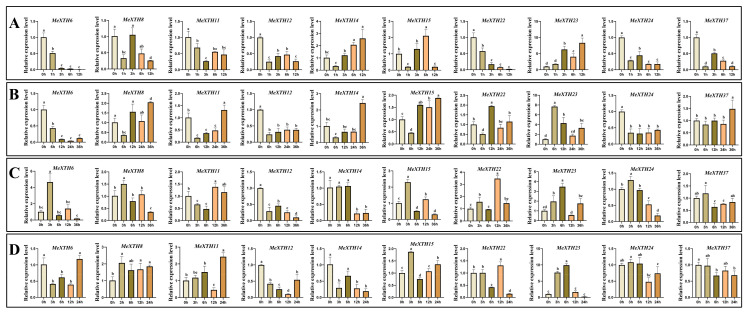
Expression analysis of the *MeXTH* gene in the leaf under abiotic stress conditions. (**A**) PEG treatment. (**B**) Salt treatment. (**C**) Heat treatment. (**D**) Cold treatment. Each bar represents the subject mean of three replicates, and error bars represent standard deviation (SD). Lowercase letters indicate differences significant at the 0.05 level.

Based on the RNA-seq expression profile, ten *MeXTH* genes with high transcriptional abundance in various tissues were selected. The quantitative real-time PCR (qRT-PCR) was used to analyze the temporal and spatial expression levels of *MeXTH* genes in different tissues (leaves, stems and roots) under abiotic stress (drought, salt, high temperature, and cold). The results demonstrate that the ten *MeXTH* genes (*MeXTH6/8/11/12/14/15/22/23/24/37*) exhibit differential but significant transcriptional responsiveness to drought, salt, and high-temperature and low-temperature treatments. (Figure 6, Figure 7 and Figure 8).

Under drought treatment, transcript levels of *MeXTH14* and *MeXTH15* in leaves were rapidly induced, peaking at 12 h and 6 h, respectively, with approximately 2-fold higher expression than the initial level, whereas other *MeXTH* genes showed gradual downregulation. In stems, expression of *MeXTH37* and *MeXTH15* strongly increased, reaching a maximum at 6 h with nearly 20-fold induction, while *MeXTH8/22/23* were upregulated by approximately 10-fold. Most other *MeXTH* genes in stems were induced to about 2-fold. In roots, expression of *MeXTH8* and *MeXTH15* displayed a typical increase-then-decrease pattern, with maximal expression at 1 h (approximately 3-fold and 6-fold, respectively). These rapid and organ-specific inductions suggest that *MeXTH* genes participate in drought-responsive cell wall loosening and reinforcement, especially in vascular and epidermal tissues, to maintain water transport capacity and cellular integrity under water deficit.

Under salt stress, *MeXTH23* was significantly upregulated in both leaves and stems, peaking at 6 h and 24 h with approximately 7-fold induction. Transcript levels of *MeXTH8/11/14/15/22/23/37* in leaves were markedly higher than in control conditions, with *MeXTH23* showing the strongest response. In stems, *MeXTH37* exhibited the highest expression, and most *MeXTH* genes were slowly and significantly upregulated, except *MeXTH6,* which was downregulated. In roots, only *MeXTH15* was slowly induced to about 2-fold, whereas all other *MeXTH* genes were gradually repressed. This organ-specific expression divergence indicates that *MeXTH*-mediated cell wall modification supports ion compartmentalization in shoots and restrains root elongation under salt stress, thereby balancing growth inhibition and stress tolerance.

Under high-temperature treatment, multiple *MeXTH* genes (*MeXTH6/8/11/15/22/23* in leaves, *MeXTH6/8/12/14/24/37* in stems, and *MeXTH6/8/11/12/14/15/23* in roots) showed an increase-then-decrease expression pattern, indicating a conserved and rapid cell wall adjustment in response to heat-induced cellular damage. Meanwhile, *MeXTH12* in leaves, *MeXTH15/22* in stems, and *MeXTH22/37* in roots were downregulated, suggesting tissue-specific fine-tuning of cell wall dynamics. Under low-temperature stress, *MeXTH23* in leaves showed the most dramatic fluctuation, peaking at 6 h with approximately 10-fold induction. In stems, most *MeXTH* genes were upregulated, except *MeXTH6/15*. In roots, however, only *MeXTH8/15/37* were induced, whereas others were downregulated, showing an expression pattern roughly opposite to that in stems. This opposite regulatory trend between roots and stems implies that *MeXTH* genes modulate tissue-specific cell wall rigidity and flexibility to cope with low-temperature-induced mechanical constraints, protecting vascular transport and cellular stability.

Collectively, the organ-specific and stress-dependent expression profiles of *MeXTH* genes demonstrate their crucial roles in cell wall remodeling during abiotic stress responses.

### 3.8. Subcellular Localization of MeXTH Protein

**Figure 9:**
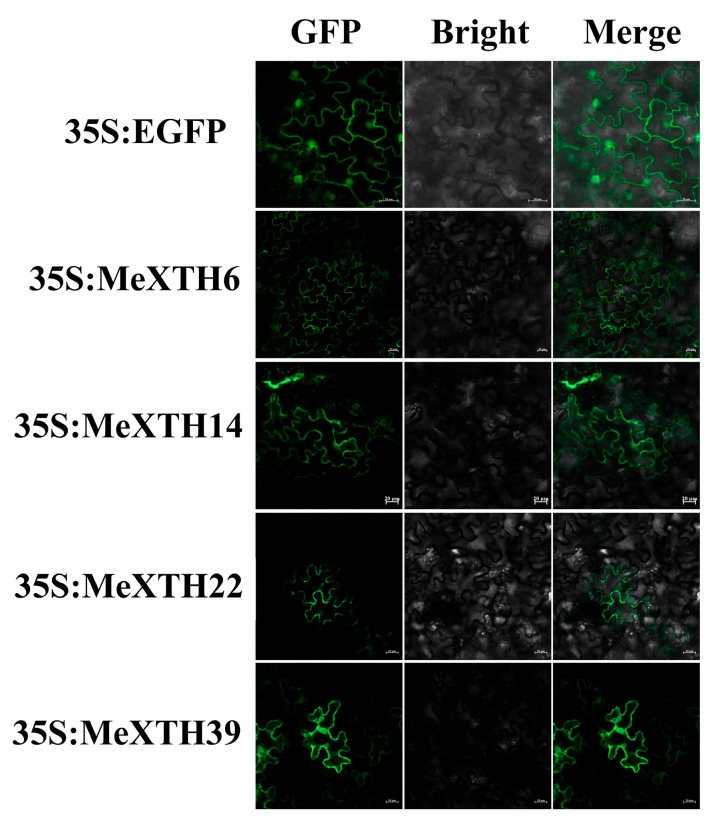
Subcellular localization of MeXTH6, 14, 22, 39.

To elucidate the subcellular localization of the MeXTH protein, this experiment constructed plant expression vectors that fused 35S:EGFP, namely 35S:MeXTH6-EGFP, 35S:MeXTH14-EGFP, 35S:MeXTH15-EGFP, 35S:MeXTH22-EGFP, and 35S:MeXTH39-EGFP. 35S:EGFP served as the empty control group, and the GFP signal produced was observable in the cell nucleus, cytoplasm, and cell margins. However, the other proteins only showed GFP signals in the cell margins (Figure 9).

**Figure 10:**
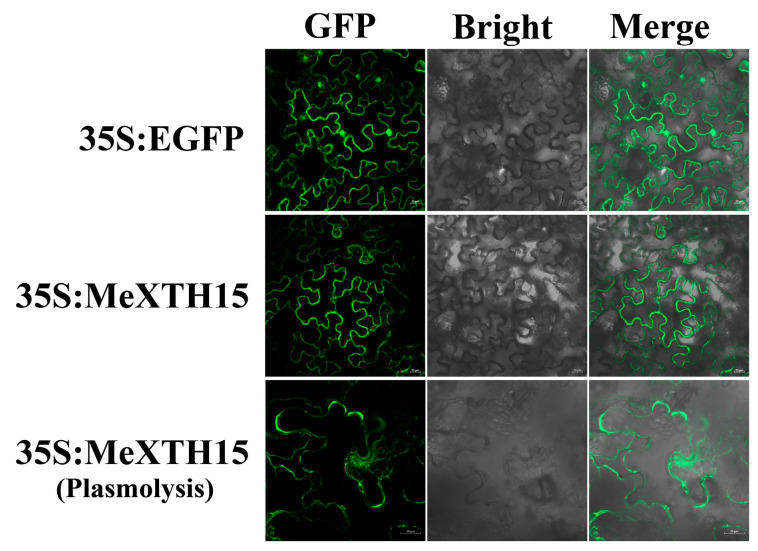
Subcellular localization of MeXTH15.

To further verify the subcellular localization of MeXTH15, we performed a plasmolysis assay. Fluorescence signals of the MeXTH15 fusion protein were clearly detected in the cell wall (Figure 10), confirming that MeXTH15 localizes to the cell wall. Accordingly, we inferred that MeXTH6, MeXTH14, MeXTH15, MeXTH22 and MeXTH39 are all cell wall-localized proteins, which is largely consistent with the predicted subcellular localization results.

## 4. Discussion

The *XTH* gene family is widespread in the plant kingdom and has been identified in a variety of plants, and the number of members of the family varies significantly in different species. For instance, *A. thaliana* contains 33 *AtXTH* genes [36], while wheat (*Triticum aestivum*) is one of the species with the largest known *XTH* gene family, having 135 *TaXTH* genes [37], sweet potato (*Ipomoea batatas*) has 36 *IbXTH* genes [38], and tea tree (*Camellia oleifera*) has 31 *CoXTH* genes [39]. A total of 42 *MeXTH* genes were identified from the whole genome of cassava, with a quantity greater than that of *A. thaliana* and sweet potato but much smaller than that of wheat. The 42 MeXTH proteins showed significant differences in amino acid quantity, molecular weight, isoelectric point, and chromosomal distribution. The distribution of *MeXTH* genes on the chromosomes of cassava is highly uneven and shows clustering characteristics. Specifically, the 42 *MeXTH* genes are unevenly distributed on 14 chromosomes, with chromosome 14 showing a highly clustered phenomenon. Similarly, 36 *XTH* genes are distributed on 13 chromosomes in sweet potato [38], and 34 *SsuXTH* genes are distributed on 18 chromosomes in *Schima superba* [40], which also show the characteristics of uneven distribution. This non-random distribution pattern indicates that chromosome structure and gene duplication may have an impact on the evolution of *XTH* family.

The phylogenetic tree was constructed using the full-length amino acid sequences of 42 cassava MeXTH proteins and 33 *A. thaliana* AtXTH proteins. The analysis of the phylogenetic tree showed that XTH proteins from cassava and *A. thaliana* can be divided into four subgroups. MeXTH members in the same group have more similar conservative structures and close genetic relationships. For example, MeXTH12, 14 and AtXTH32 were highly similar, and MeXTH6/7/12/14 were highly similar to AtXTH31/32. The analysis of conserved motifs showed that almost all MeXTH proteins (except MeXTH2, MeXTH6, and MeXTH17) contained these conserved motifs in a consistent order. The difference was that the N-terminus of MeXTH2 did not have Motif 10 or Motif 6, and the C-terminus did not contain Motif 5 and Motif 9. Meanwhile, the C-terminus of MeXTH6 and MeXTH17 lacked Motif 9, indicating that some MeXTH members might have lost the C-terminal or N-terminal during evolution. Except for MeXTH1, which contains only one exon, the genetic structure of MeXTH can roughly be divided into three patterns: containing two exons and one intron, three exons and two introns, and four exons and three introns. Among them, the number of genes containing four exons and three introns is the largest, reaching twenty. It accounts for nearly half of the number of the MeXTH gene family. In plants, the evolution of exon–intron structure is closely related to functional adaptability. The structure between exons and introns directly or indirectly affects gene expression regulation and the efficiency of mRNA alternative splicing. Genes with more exons may produce more proteins after alternative splicing, enabling plants to better adapt to changes in the external environment [41]. Most cassava MeXTH proteins may be generated through alternative splicing after experiencing changes in the external environment. The MeXTH gene family is divided into four subfamilies, including Group I/II, Group IIIA, Group IIIB, and the Ancestral Group. Members of the Ancestral group exhibit extensive substrate specificity and can act not only on xyloglucan but also hydrolyze cellulose analogues such as hydroxyethyl cellulose and linked β-glucans. They may have had cell wall modification functions in the early stages of evolution [42]. The members of the Group I/II and the Group IIIA/IIIB are more involved in organ-specific development such as fruit softening, root elongation, and responses to drought, salt, and heavy metal stress. Their expression patterns are regulated by stress-responsive elements in the promoter region such as ABRE, DRE, and hormone-responsive elements such as MeJA and ABA [43,44,45]. The results of phylogenetic tree analysis indicate that the evolutionary relationship of the MeXTH gene family presents a complex and orderly pattern. This classification system is highly conserved in cassava and *A. thaliana*, suggesting that functional differentiation occurred in the early stages of evolution [42,46].

A large number of studies have shown that gene duplication events, including tandem duplication, segmental duplication, and whole-genome duplication, are the main driving forces for the evolution of gene families [47,48]. The *MeXTH* gene family mainly expands in the cassava genome through segmental duplication events, while in wheat (*T. aestivum*), 135 *TaXTH* genes, in poplar (*Populus trichocarpa*), 43 *PtrXTH* genes, and in barley (*Hordeum vulgare*), 24 *HvXTH* genes, the expansion is mainly through both tandem duplication and segmental duplication patterns [37,49,50]. This expansion pattern leads to the redundancy and diversity of gene functions, while also preserving the core structural features. As non-coding DNA sequences in the genome, cis-acting elements play a crucial role in gene transcription regulation and are often regarded as molecular switches for gene expression regulation [51]. Many elements related to light response, hormone response and stress response, as well as those related to plant growth and development, have been found in the promoter region of the *MeXTH* genes. However, the cis-acting elements in each *MeXTH* gene promoter are different. For instance, 25 *MeXTH* genes contained anaerobic response elements (ARE), 19 genes contained MYB binding sites involved in drought stress (MBS), 14 genes contained low-temperature response elements (LTR), 30 genes contained MeJA-responsive motifs (TGACG-motif), 30 genes contained abscisic acid-responsive elements (ABRE), 11 genes contained salicylic acid-responsive elements (TCA-element), and 12 genes contained auxin-responsive elements (TGA-element). In addition, Strigolactones are indispensable carotenoid-derived phytohormones that widely participate in the regulation of plant stress adaptation, cell wall remodeling, and root morphological development under adverse conditions. Previous studies have demonstrated that strigolactone signaling substantially modulates the expression of numerous cell wall-related genes, thereby coordinating cell wall flexibility and structural stability during abiotic stress responses in tropical crops [52,53]. In the present study, multiple *MeXTH* genes exhibited significantly altered expression levels under abiotic stress, implying that dynamic cell wall reconstruction occurred in stressed cassava. Combined with published evidence, we speculate that strigolactone signals may participate in the upstream regulation of *MeXTH*-mediated cell wall modification. Similar regulatory patterns have been reported in many crops, in which strigolactone fine-tunes cell wall extensibility to mitigate stress-induced cell damage [54,55]. Therefore, strigolactone-mediated hormonal regulation is considered one of the potential pathways assisting cassava to adapt to hostile abiotic environments. These results suggest that the *MeXTH* genes in cassava may have multiple or specific functions and may play a key role in regulating cassava growth, development, and responses to various environmental stresses.

The *MeXTH* gene exhibits a complex regulatory network in response to abiotic stresses (drought stress, salt stress, high-temperature stress, and low-temperature stress). The results show that *MeXTH24* only responds to high-temperature stress, while *MeXTH14/22/23/37* can respond simultaneously to three stresses (drought, salt, and high-temperature), and *MeXTH8/11/15* can respond to all four stresses. This indicates that they may play a key role in regulating various stress-related responses. Furthermore, the changes in gene expression levels and time during different treatment processes do not show a simple linear relationship. The timeliness of expression of different genes under abiotic stress conditions varies, and the expression patterns of the same gene in different tissues and under different abiotic stresses are also completely different. These results indicate that the regulation of the *MeXTH* gene family in response to abiotic stress is a complex process. The *MeXTH* gene family may play a functional role in abiotic stress responses, which has also been confirmed by other studies. For example, under drought stress, overexpression of *CoXTH1* in tea plants enhances cell wall stability by increasing the contents of cellulose, hemicellulose, and xyloglucan, while also increasing antioxidant enzyme activity to maintain oxidative balance, thereby enhancing the drought resistance of *A. thaliana* plants [39]. Banana *MaXTH7/8* and sweet potato *IbXTH16* enhance the cold resistance of plants by activating the BR signaling pathway and proline synthesis [56,57]. The *AtXTH19* mutant of *A. thaliana* has abnormal distribution of cell wall xyloglucan after cold damage, and its cold resistance is reduced [58]. Under aluminum (Al^3+^) stress, the heterologous expression of *ZmXTH* in *A. thaliana* plants reduces the aluminum content in the cell walls of *A. thaliana* roots, thereby weakening the toxicity of aluminum to *A. thaliana* [59]. Similarly, overexpression of *OsXTH19* in *A. thaliana* can also enhance the aluminum resistance of *A. thaliana* [60].

Although these findings indicate that the *MeXTH* gene exhibits a measurable and stress-responsive expression pattern under abiotic stress conditions, more in-depth research is still needed to fully understand this adverse response mechanism. The evolution of the *MeXTH* gene family is closely related to the growth, development and environmental adaptation of cassava. From its fundamental role in the dynamic remodeling of cell walls to its special functions in response to abiotic stress in cassava, the *MeXTH* gene provides a molecular basis for cassava to adapt to complex and changeable environments through diverse expression patterns.

## 5. Conclusions

This study is the first to identify 42 *MeXTH* genes from the whole genome of cassava, and investigate their phylogeny, gene structure, conserved motifs, chromosomal distribution, intraspecific and interspecific collinearity, cis-acting elements, and subcellular localization. Additionally, quantitative real-time PCR analysis results show that the selected ten members of the *MeXTH* gene family can all be significantly induced by at least one of the stress conditions such as drought, salt, high temperature, and low temperature, revealing the unique spatiotemporal expression pattern of *MeXTH* genes under abiotic stress, and filling the key gap in current cassava research. In conclusion, the cassava *MeXTH* gene family may play a key role in abiotic stress responses, laying the foundation for further clarifying the function of the *MeXTH* gene in abiotic responses.

## Data Availability

The RNA-seq data used in this study are available in the NCBI database (GEO dataset: GSE82279 and GSE156638). Additional data supporting the findings can be found in the Supplementary Materials.

## Supplementary Materials

The following supporting information can be downloaded at https://www.mdpi.com/article/10.3390/genes17060613/s1, Table S1: Basic information and Subcellular localization of cassava XTH proteins. Table S2: Information on the primers used in the experiment.

## References

[1] Olsen K., Schaal B. (2001). Microsatellite variation in cassava (*Manihot esculenta*, Euphorbiaceae) and its wild relatives: Further evidence for a southern Amazonian origin of domestication. Am. J. Bot..

[2] Wilson M.C., Mutka A.M., Hummel A.W., Berry J., Chauhan R.D., Vijayaraghavan A., Taylor N.J., Voytas D.F., Chitwood D.H., Bart R.S. (2017). Gene expression atlas for the food security crop cassava. New Phytol..

[3] Anokye B., Amoah P., Potter B.W., Mahamane A.-R.O., Adu-Gyamfi T., Dembure L., Abadura N.A., Olasanmi B., Parkes E. (2026). Genetic diversity assessment of hydrogen cyanide, total carotenoid content, and dry matter content in biofortified cassava using trait-linked SNP markers. Czech J. Genet. Plant Breed..

[4] Zhu J.K. (2016). Abiotic Stress Signaling and Responses in Plants. Cell.

[5] Zárate-Chaves C.A., Gómez de la Cruz D., Verdier V., López C.E., Bernal A., Szurek B. (2021). Cassava diseases caused by *Xanthomonas phaseoli* pv. *manihotis* and *Xanthomonas cassavae*. Mol. Plant Pathol..

[6] Xiao L., Cheng D., Ou W., Chen X., Rabbi I.Y., Wang W., Li K., Yan H. (2024). Advancements and strategies of genetic improvement in cassava (*Manihot esculenta* Crantz): From conventional to genomic approaches. Hortic. Res..

[7] Hong Y., Xiao Y., Song N., Zhu S., Zhao R., Li K., Geng M., Yu X., Wang H., Xia W. (2021). Identification and characterization of MeERF genes and their targets in pathogen response by cassava (*Manihot esculenta*). Crop J..

[8] Cosgrove D.J. (2024). Structure and growth of plant cell walls. Nat. Rev. Mol. Cell Biol..

[9] Zhang B., Gao Y., Zhang L., Zhou Y. (2021). The plant cell wall: Biosynthesis, construction, and functions. J. Integr. Plant Biol..

[10] Rose J.K., Braam J., Fry S.C., Nishitani K. (2002). The XTH family of enzymes involved in xyloglucan endotransglucosylation and endohydrolysis: Current perspectives and a new unifying nomenclature. Plant Cell Physiol..

[11] Hara Y., Yokoyama R., Osakabe K., Toki S., Nishitani K. (2013). Function of xyloglucan endotransglucosylase/hydrolases in rice. Ann. Bot..

[12] Saladié M., Rose J.K., Cosgrove D.J., Catalá C. (2006). Characterization of a new xyloglucan endotransglucosylase/hydrolase (XTH) from ripening tomato fruit and implications for the diverse modes of enzymic action. Plant J..

[13] Miedes E., Suslov D., Vandenbussche F., Kenobi K., Ivakov A., Van Der Straeten D., Lorences E.P., Mellerowicz E.J., Verbelen J.P., Vissenberg K. (2013). Xyloglucan endotransglucosylase/hydrolase (XTH) overexpression affects growth and cell wall mechanics in etiolated *Arabidopsis* hypocotyls. J. Exp. Bot..

[14] Zhai Z., Feng C., Wang Y., Sun Y., Peng X., Xiao Y., Zhang X., Zhou X., Jiao J., Wang W. (2021). Genome-Wide Identification of the Xyloglucan endotransglucosylase/Hydrolase (XTH) and Polygalacturonase (PG) Genes and Characterization of Their Role in Fruit Softening of Sweet Cherry. Int. J. Mol. Sci..

[15] Muñoz-Bertomeu J., Miedes E., Lorences E.P. (2013). Expression of xyloglucan endotransglucosylase/hydrolase (XTH) genes and XET activity in ethylene treated apple and tomato fruits. J. Plant Physiol..

[16] Zhang Z., Wang N., Jiang S., Xu H., Wang Y., Wang C., Li M., Liu J., Qu C., Liu W. (2017). Analysis of the Xyloglucan Endotransglucosylase/Hydrolase Gene Family during Apple Fruit Ripening and Softening. J. Agric. Food Chem..

[17] Ran F., Xiang C., Wang C., Zang Y., Liu L., Wu S., Wang C., Cai J., Wang D., Min Y. (2024). Identification of the 4CL family in cassava (*Manihot esculenta* Crantz) and expression pattern analysis of the Me4CL32 gene. Biochem. Biophys. Res. Commun..

[18] Li S., Yu X., Cheng Z., Yu X., Ruan M., Li W., Peng M. (2017). Global Gene Expression Analysis Reveals Crosstalk between Response Mechanisms to Cold and Drought Stresses in Cassava Seedlings. Front. Plant Sci..

[19] Lei N., Yu X., Li S., Zeng C., Zou L., Liao W., Peng M. (2017). Phylogeny and expression pattern analysis of TCP transcription factors in cassava seedlings exposed to cold and/or drought stress. Sci. Rep..

[20] Yu F., Lin C., Xie X., Yu X., Guo X. (2025). Genome-Wide Identification and Expression Analysis of CAMTA Genes in Cassava Under Abiotic Stresses. Plants.

[21] Chen C., Chen H., Zhang Y., Thomas H.R., Frank M.H., He Y., Xia R. (2020). TBtools: An Integrative Toolkit Developed for Interactive Analyses of Big Biological Data. Mol. Plant.

[22] Wilkins M.R., Gasteiger E., Bairoch A., Sanchez J.C., Williams K.L., Appel R.D., Hochstrasser D.F. (1999). Protein identification and analysis tools in the ExPASy server. Methods Mol. Biol..

[23] Chou K.C., Shen H.B. (2008). Cell-PLoc: A package of Web servers for predicting subcellular localization of proteins in various organisms. Nat. Protoc..

[24] Bailey T.L., Boden M., Buske F.A., Frith M., Grant C.E., Clementi L., Ren J., Li W.W., Noble W.S. (2009). MEME SUITE: Tools for motif discovery and searching. Nucleic Acids Res..

[25] Tamura K., Stecher G., Kumar S. (2021). MEGA11: Molecular Evolutionary Genetics Analysis Version 11. Mol. Biol. Evol..

[26] Kumar S., Gadagkar S.R. (2000). Efficiency of the neighbor-joining method in reconstructing deep and shallow evolutionary relationships in large phylogenies. J. Mol. Evol..

[27] Kumar S., Stecher G., Li M., Knyaz C., Tamura K. (2018). MEGA X: Molecular Evolutionary Genetics Analysis across Computing Platforms. Mol. Biol. Evol..

[28] Wang Y., Tang H., Debarry J.D., Tan X., Li J., Wang X., Lee T.H., Jin H., Marler B., Guo H. (2012). MCScanX: A toolkit for detection and evolutionary analysis of gene synteny and collinearity. Nucleic Acids Res..

[29] Lescot M., Déhais P., Thijs G., Marchal K., Moreau Y., Van de Peer Y., Rouzé P., Rombauts S. (2002). PlantCARE, a database of plant cis-acting regulatory elements and a portal to tools for in silico analysis of promoter sequences. Nucleic Acids Res..

[30] Sullivan D.K., Min K.H.J., Hjörleifsson K.E., Luebbert L., Holley G., Moses L., Gustafsson J., Bray N.L., Pimentel H., Booeshaghi A.S. (2025). kallisto, bustools and kb-python for quantifying bulk, single-cell and single-nucleus RNA-seq. Nat. Protoc..

[31] Morante-Carriel J., Sellés-Marchart S., Martínez-Márquez A., Martínez-Esteso M.J., Luque I., Bru-Martínez R. (2014). RNA isolation from loquat and other recalcitrant woody plants with high quality and yield. Anal. Biochem..

[32] Mo C., Wan S., Xia Y., Ren N., Zhou Y., Jiang X. (2018). Expression Patterns and Identified Protein-Protein Interactions Suggest That Cassava CBL-CIPK Signal Networks Function in Responses to Abiotic Stresses. Front. Plant Sci..

[33] Livak K.J., Schmittgen T.D. (2001). Analysis of relative gene expression data using real-time quantitative PCR and the 2^−ΔΔ*C*T^ Method. Methods.

[34] Court D.L., Sawitzke J.A., Thomason L.C. (2002). Genetic Engineering Using Homologous Recombination. Annu. Rev. Genet..

[35] Segatto R., Jones T., Stretch D., Albin C., Chauhan R.D., Taylor N.J. (2022). Agrobacterium-mediated Genetic Transformation of Cassava. Curr. Protoc..

[36] Yokoyama R., Nishitani K. (2001). A comprehensive expression analysis of all members of a gene family encoding cell-wall enzymes allowed us to predict cis-regulatory regions involved in cell-wall construction in specific organs of *Arabidopsis*. Plant Cell Physiol..

[37] Bi H., Liu Z., Liu S., Qiao W., Zhang K., Zhao M., Wang D. (2024). Genome-wide analysis of wheat xyloglucan endotransglucosylase/hydrolase (XTH) gene family revealed TaXTH17 involved in abiotic stress responses. BMC Plant Biol..

[38] Zhang J.Z., He P.W., Xu X.M., Lü Z.F., Cui P., George M.S., Lu G.Q. (2023). Genome-Wide Identification and Expression Analysis of the Xyloglucan Endotransglucosylase/Hydrolase Gene Family in Sweet Potato [*Ipomoea batatas* (L.) Lam]. Int. J. Mol. Sci..

[39] Ma Y., Zhang Y., Zhang Z., He Z., Xun C., Wang X., Zhang Y., Wang R., Chen Y. (2025). Genome-Wide Identification and Characterization of the Xyloglucan Endotransglucosylase/Hydrolase (XTH) Gene Family in Camellia oleifera and the Function of CoXTH1 During Drought Stress. Plants.

[40] Yang Z., Zhang R., Zhou Z. (2022). The XTH Gene Family in Schima superba: Genome-Wide Identification, Expression Profiles, and Functional Interaction Network Analysis. Front. Plant Sci..

[41] Laloum T., Martín G., Duque P. (2018). Alternative Splicing Control of Abiotic Stress Responses. Trends Plant Sci..

[42] Seven M., Derman Ü.C., Harvey A.J. (2021). Enzymatic characterization of ancestral/group-IV clade xyloglucan endotransglycosylase/hydrolase enzymes reveals broad substrate specificities. Plant J..

[43] Ishida K., Yokoyama R. (2022). Reconsidering the function of the xyloglucan endotransglucosylase/hydrolase family. J. Plant Res..

[44] Qiao T., Zhang L., Yu Y., Pang Y., Tang X., Wang X., Li L., Li B., Sun Q. (2022). Identification and expression analysis of xyloglucan endotransglucosylase/hydrolase (XTH) family in grapevine (*Vitis vinifera* L.). PeerJ.

[45] Han T., Cui Y., Jing Y., Liu M., Chen X., Song Y., Gu X., Wang J., Wang L. (2025). Xyloglucan endotransglucosylase/hydrolase 25 positively regulates the lead tolerance in Raphanus sativus. Front. Plant Sci..

[46] Del-Bem L.E. (2018). Xyloglucan evolution and the terrestrialization of green plants. New Phytol..

[47] Xie T., Chen C., Li C., Liu J., Liu C., He Y. (2018). Genome-wide investigation of WRKY gene family in pineapple: Evolution and expression profiles during development and stress. BMC Genom..

[48] Wang R., Zhao P., Kong N., Lu R., Pei Y., Huang C., Ma H., Chen Q. (2018). Genome-Wide Identification and Characterization of the Potato bHLH Transcription Factor Family. Genes.

[49] Cheng Z., Zhang X., Yao W., Gao Y., Zhao K., Guo Q., Zhou B., Jiang T. (2021). Genome-wide identification and expression analysis of the xyloglucan endotransglucosylase/hydrolase gene family in poplar. BMC Genom..

[50] Fu M.M., Liu C., Wu F. (2019). Genome-Wide Identification, Characterization and Expression Analysis of Xyloglucan Endotransglucosylase/Hydrolase Genes Family in Barley (*Hordeum vulgare*). Molecules.

[51] Cui X., Yin Q., Gao Z., Li Z., Chen X., Lv H., Chen S., Liu Q., Zeng W., Jiang R. (2025). CREATE: Cell-type-specific cis-regulatory element identification via discrete embedding. Nat. Commun..

[52] Khan M.K., Pandey A., Hamurcu M., Vyhnanek T., Zargar S.M., Kahraman A., Topal A., Gezgin S. (2024). Exploring strigolactones for inducing abiotic stress tolerance in plants. Czech J. Genet. Plant Breed..

[53] Dong J., Fu H., Wang Z., Zhang L., Liu Z., Hu Y., Shen F., Wang W. (2025). Mechanisms of Strigolactone-Regulated Abiotic Stress Responses in Plants. Plants.

[54] Sun H., Tao J., Gu P., Xu G., Zhang Y. (2016). The role of strigolactones in root development. Plant Signal. Behav..

[55] Koltai H. (2011). Strigolactones are regulators of root development. New Phytol..

[56] Tan Y., Zhan H., Chen H., Li X., Chen C., Liu H., Chen Y., Zhao Z., Xiao Y., Liu J. (2024). Genome-wide identification of XTH gene family in Musa acuminata and response analyses of MaXTHs and xyloglucan to low temperature. Physiol. Plant..

[57] Yu T., Pan J., Liu S., Yang Z., Liu Z. (2025). A xyloglucan endotransglucosylase/hydrolase gene, IbXTH16, increases cold tolerance in transgenic sweetpotato. Front. Genet..

[58] Takahashi D., Johnson K.L., Hao P., Tuong T., Erban A., Sampathkumar A., Bacic A., Livingston D.P., Kopka J., Kuroha T. (2021). Cell wall modification by the xyloglucan endotransglucosylase/hydrolase XTH19 influences freezing tolerance after cold and sub-zero acclimation. Plant Cell Environ..

[59] Du H., Hu X., Yang W., Hu W., Yan W., Li Y., He W., Cao M., Zhang X., Luo B. (2021). ZmXTH, a xyloglucan endotransglucosylase/hydrolase gene of maize, conferred aluminum tolerance in *Arabidopsis*. J. Plant Physiol..

[60] Tatsumi A., Nagayama T., Teramoto A., Nakamura A., Yokoyama R., Furukawa J., Iwai H. (2025). OsXTH19 Overexpression Improves Aluminum Tolerance via Xyloglucan Reduction in Rice Root Cell Wall. Plants.

