# Supplementary material for: Genome-Wide Identification and Expression Analysis of the Xyloglucan Endotransglucosylase/Hydrolase Gene Family in Manihot esculenta
Source: Genes (Basel). 2026 May 29;17(6):613. doi: 10.3390/genes17060613 (PMC13298684; doi:10.3390/genes17060613)
Supplement: Supplementary file 1 [file genes-17-00613-s001.zip › genes-4277227-supplementary.pdf]

Table S1. Basic information and Subcellular localization of cassava XTH proteins.

Name	Gene ID	Number of Amino Acid (aa)	Molecular Weight (Da)	Theoretical pI	Number of negatively charged residues	Number of positively charged residues	Instability index	Subcellular Localization
MeXTH1	Manes.01G241900	286	32808.8	5.75	32	26	44.19	Cell wall
MeXTH2	Manes.01G262300	262	29619.21	4.93	37	22	44.56	Cell wall
MeXTH3	Manes.03G024400	294	33601.86	8.53	24	27	42.45	Cell wall
MeXTH4	Manes.03G079700	287	32554.98	9.04	22	28	43.33	Cell wall
MeXTH5	Manes.03G146800	290	33136.98	7.14	26	26	47.38	Cell wall
MeXTH6	Manes.04G013900	290	33002.81	5.67	29	24	43.10	Cell wall
MeXTH7	Manes.04G049900	279	31318.92	4.55	26	15	34.81	Cell wall,Cytoplasm
MeXTH8	Manes.04G096600	293	34042.43	8.45	32	35	45.54	Cell wall,Cytoplasm
MeXTH9	Manes.05G108100	290	32505.58	6.14	26	25	33.8	Cell wall,Cytoplasm
MeXTH10	Manes.05G199600	293	33090.42	8.65	26	30	42.41	Cell wall,Cytoplasm
MeXTH11	Manes.07G051100	294	34457.98	8.59	29	32	36.91	Cell wall
MeXTH12	Manes.08G011900	326	37624.88	9.40	22	34	50.85	Cell wall
MeXTH13	Manes.08G099000	293	34103.64	8.47	31	34	40.76	Cell wall,Cytoplasm
MeXTH14	Manes.09G064800	294	33989.53	9.42	23	34	45.88	Cell wall
MeXTH15	Manes.11G072700	293	34014.33	6.16	32	29	40.49	Cell wall,Cytoplasm
MeXTH16	Manes.11G115200	284	32091.14	8.24	23	25	23.06	Cell wall,Cytoplasm
MeXTH17	Manes.11G151500	291	33141.18	7.66	26	27	39.38	Cell wall
MeXTH18	Manes.12G030000	315	35414.09	6.40	28	25	56.91	Cell wall
MeXTH19	Manes.12G076800	288	32484.49	5.48	33	22	36.66	Cell wall
MeXTH20	Manes.12G118804	288	33203.59	7.63	29	30	39.59	Cell wall
MeXTH21	Manes.13G046500	296	33897.19	5.77	26	21	32.21	Cell wall
MeXTH22	Manes.13G107100	292	33454.84	6.38	30	28	51.52	Cell wall
MeXTH23	Manes.14G114400	336	38143.11	6.99	29	29	31.63	Cell wall,Cytoplasm
MeXTH24	Manes.14G114500	293	33554.72	5.69	29	24	33.53	Cell wall,Cytoplasm
MeXTH25	Manes.14G120866	285	32231.02	5.09	26	21	33.21	Cell wall,Cytoplasm
MeXTH26	Manes.14G127232	322	36605.26	5.82	27	24	36.90	Cell wall,Cytoplasm
MeXTH27	Manes.14G133598	266	30196.47	5.07	26	21	33.86	Cell wall,Cytoplasm
MeXTH28	Manes.14G139964	322	36563.17	5.83	27	24	36.30	Cell wall,Cytoplasm
MeXTH29	Manes.14G145700	280	31707.43	5.22	26	21	33.97	Cell wall,Cytoplasm
MeXTH30	Manes.14G145800	280	31707.43	5.22	26	21	33.97	Cell wall,Cytoplasm
MeXTH31	Manes.14G146000	285	32299.05	4.95	26	19	33.74	Cell wall,Cytoplasm
MeXTH32	Manes.14G146100	285	32277.99	4.94	26	20	36.14	Cell wall,Cytoplasm
MeXTH33	Manes.14G146333	322	36521.16	5.82	27	24	36.86	Cell wall,Cytoplasm
MeXTH34	Manes.14G146933	285	32231.02	5.09	26	21	33.21	Cell wall,Cytoplasm
MeXTH35	Manes.14G152100	283	31942.9	8.12	24	26	31.74	Cell wall,Cytoplasm
MeXTH36	Manes.14G152200	286	32482.46	6.89	22	22	37.17	Cell wall,Cytoplasm
MeXTH37	Manes.15G137800	333	37979.67	5.94	42	36	49.03	Cell wall
MeXTH38	Manes.15G192300	342	39685.97	9.04	35	45	47.83	Cell wall
MeXTH39	Manes.16G011700	297	34726.87	5.00	40	29	39.38	Cell wall
MeXTH40	Manes.17G015100	348	40103.5	9.17	37	47	46.91	Cell wall
MeXTH41	Manes.17G063600	295	33822.16	8.72	28	32	36.60	Cell wall,Cytoplasm
MeXTH42	Manes.17G087100	332	37735.4	7.14	36	36	50.22	Cell wall

Table S2. Information on the primers used in the experiment

Primer name	Primers sequence（5'- 3'）
qMeXTH6-F	5'-TCAAGCACCACTGCTCAAAA-3'
qMeXTH6-R	5'-ATGTGCCTTGGTCTAGCCTTT-3'
qMeXTH8-F	5'-CTGTGCCTGAGTTTCCTCCTT-3'
qMeXTH8-R	5'-AGCCCATGTTGGTGCATAGT-3'
qMeXTH11-F	5'-TTGGGAATGTGAGTGGGCAG-3'
qMeXTH11-R	5'-GGTGAAGGTTCCACAAGACAGA-3'
qMeXTH12-F	5'-CTTTCTTCTATCTCTCTCTCCCCC-3'
qMeXTH12-R	5'-AGTAGCAAGAGAAGGAGAGCC-3'
qMeXTH14-F	5'-GCCCTCTTCCTCTTACTTCTTCT-3'
qMeXTH14-R	5'-TGATGTGTGTAACCCCAGAGA-3'
qMeXTH15-F	5'-AGCGTTGGTGGGATCAGAAG-3'
qMeXTH15-R	5'-TCACGCTGGCATTCAGGAG-3'
qMeXTH22-F	5'-GCAAATTGTGCAGCAAACCC-3'
qMeXTH22-R	5'-ACTTGTCGGTGCAGTAGTCG-3'
qMeXTH23-F	5'-ATTGGGCTACAAGGGGTGGT-3'
qMeXTH23-R	5'-TGCTGTTAGAGGCAGAAGTTG-3'
qMeXTH24-F	5'-AACAAGTCAGGAGAGGCTGAAA-3'
qMeXTH24-R	5'-ATGATGGAGCCAAGACATGAT-3'
qMeXTH37-F	5'-GTCTGGGTTCGTTTCTCAGGAC-3'
qMeXTH37-R	5'-TAAAAAGCGACCACAACTCCA-3'
pSCZ-MeXTH6F	5'-taattaagacccgggacATGGCTTCTCTGCTTTATCTTCTGC-3'
pSCZ-MeXTH6R	5'-agcaggactctagggTTAACATTCAGGTGTTTGGGTATGA-3'
pSCZ-MeXTH14F	5'-taattaagacccgggacATGGCCCTCTTCCTCTTACTTCTT-3'
pSCZ-MeXTH14R	5'-agcaggactctagggTTAACGCCGACACTCGGG-3'
pSCZ-MeXTH15F	5'-taattaagacccgggacATGGCTTATTTTCTATGGACTCTATGC-3'
pSCZ-MeXTH15R	5'-agcaggactctagggTCAAATGTCGTGGTCACGCTG-3'
pSCZ-MeXTH22F	5'-taattaagacccgggacATGGCCTTGTGTCTCTCCTTAAGA-3'
pSCZ-MeXTH22R	5'-agcaggactctagggTCAGACCCCGGCCATGCA-3'
pSCZ-MeXTH39F	5'-taattaagacccgggacATGGATTGCTGTCTCTCGTCTCTC-3'
pSCZ-MeXTH39R	5'-agcaggactctagggTTAGGACCACATAATATGATTATCATTACC-3'
MeActin-F	5'-GCCTCCCAAGGTAGCTTTCA-3'
MeActin-R	5'-GGTTAATGCAGGGCTCCACT-3'
pSCZ-speI-F	5'-AGAAGCGCGATCACATGG-3'
pSCZ-speI-R	5'-TCGGAGCTACACATGCTC-3'
